# Effect of age on the efficacy and safety of *Panax notoginseng* saponins in acute ischemic stroke: a prespecified secondary analysis of the PANDA study

**DOI:** 10.1186/s13020-025-01101-5

**Published:** 2025-04-15

**Authors:** Tingting Li, Anxin Wang, Xiaoli Zhang, Luda Feng, Peipei Du, Ying Gao, Xunming Ji, Haiqing Song, Chi Zhang

**Affiliations:** 1 Department of Neurology, Dongzhimen Hospital, Beijing University of Chinese Medicine, Beijing, 100700 China; 2 National Center for Integrative Medicine; Department of Proctology, China-Japan Friendship Hospital, Beijing, 100029 China; 3 Department of Neurology, Tiantan Hospital, Capital Medical University, Beijing, 100070 China; 4 Department of Nephrology, Dongfang Hospital, Beijing University of Chinese Medicine, Beijing, 100078 China; 5 Department of Neurology, Xuanwu Hospital, Capital Medical University, Beijing, 100053 China

**Keywords:** Acute ischemic stroke, Age, *Panax notoginseng* saponins, Prespecified analysis

## Abstract

**Background:**

The therapeutic utility of *Panax notoginseng* saponins (Xuesaitong soft capsules) for patients with acute ischemic stroke (AIS) was previously demonstrated through the PANax notoginseng Saponins Treatment of aDults with ischemic stroke in ChinA (PANDA) trial, revealing significant gains in functional independence compared to placebo. However, the related variation of older age accepted as the predictors of poor outcome, in response to *Panax notoginseng* saponins remains unexplored.

**Methods:**

We conducted a prespecified analysis of the PANDA trial to evaluate the effect of age on the efficacy and safety of Xuesaitong soft capsules. A multivariable logistic and Cox regression analysis with an interaction term was used to determine whether age (< 65 years vs. ≥ 65 years) affected the treatment effect. The primary outcome of this study was functional independence at the 3-month follow-up, as indicated by a modified Rankin Scale score (mRS) ranging from 0 to 2.

**Results:**

Between July 1 th, 2018, and June 30 th, 2020, a total of 3072 patients were recruited from 67 medical centers in China. Of these, 2966 patients were incorporated into the intention-to-treat (ITT) analysis and subsequently categorized into two age-based subgroups: (1) 1788 patients (60.28%) aged less than 65 years and (2) 1178 patients (39.72%) aged 65 years or older. Age significantly influenced the proportion of AIS patients attaining functional independence within three months [aged ≥ 65 years, adjusted odds ratio (aOR): 3.15, 95% CI: 2.13–4.67, *P* < 0.0001; aged < 65 years, aOR: 1.84, 95% CI: 1.33–2.54, *P* = 0.0002; *P* for interaction = 0.027]. Notably, a significant interaction was detected between age categories and treatment, with a greater likelihood of achieving functional independence among AIS patients aged ≥ 65 years. Regarding the primary safety outcome, which measured the rate of serious adverse events (SAEs) at 3 months, no significant difference was detected between the treatment and placebo groups across both age categories (aged ≥ 65 years, aOR: 0.32, 95% CI: 0.06–1.69, *P* = 0.181; aged < 65 years, aOR: 1.76, 95% CI: 0.41–7.47, *P* = 0.444; *P* for interaction = 0.132).

**Conclusions:**

This prespecified secondary analysis suggests that AIS patients can potentially benefit from Xuesaitong treatment in achieving functional independence, irrespective of age. Furthermore, older individuals may experience more substantial clinical benefits from Xuesaitong soft capsules for AIS.

## Introduction

Stroke ranks as the second leading cause of mortality worldwide and places a substantial public health and economic strain on China’s already burdened healthcare system [1, 2]. The principal driver of increasing stroke incidence and prevalence is rapid population aging. Ischemic stroke (IS) is the most prevalent and is particularly linked to aging. It predominantly affects middle-aged and older individuals, constituting 80% of all stroke cases [3]. The prevalence of stroke doubles with each decade of life beyond the age of 55, and a significant proportion, ranging from 75 to 89%, occurs in individuals 65 years and older [4]. Advanced age remains a major determinant of stroke risk, correlating with poorer functional outcomes and decline [5]. Older patients with IS demonstrate more adverse dysfunction and diminished quality of life compared to younger patients. Moreover, the older population is often underrepresented in clinical trials, which may result in suboptimal treatment in practical settings [6].

Although reperfusion therapies such as intravenous thrombolysis and endovascular treatment serve as the cornerstone for acute ischemic stroke (AIS) management, these interventions show limited efficacy in the geriatric AIS population (those aged > 65 years) who are frail. In this group, rtPA treatment alone or in combination with thrombectomy correlates with less improvement in neurological deficits, increased occurrence of deep venous thrombosis, and higher mortality rates [7, 8]. Although neuroprotection has been increasingly recognized as a promising therapeutic strategy in the management of AIS, the recent analyses of predefined subgroups in multiple rigorously conducted studies on the efficacy of neuroprotective agents in treating AIS revealed no significant differential benefit in older patients compared to their younger counterparts [9, 10]. Specifically, the efficacy of butylphthalide compared to a placebo, was found to be similar across both age cohorts—AIS patients over 60 years and those under 60 years—in reaching functional independence (90-day mRS score of 0–2) [10]. Aspirin’s role in the treatment and prevention of IS, owing to its antiplatelet properties, is well-established. Nonetheless, aspirin is associated with upper gastrointestinal mucosal damage, which may lead to severe complications such as ulcer bleeding. This risk is elevated in the older adults due to declining heart, kidney, and liver function [11]. Therefore, it is imperative and urgent to explore and advance the development and adoption of alternative therapeutic agents that are both effective and safe to improve the overall prognosis for the geriatric IS population.

*Panax notoginseng* saponins constitute the primary bioactive components of *Panax notoginseng* (a renowned and highly valued plant employed in Chinese medicine) and have been traditionally employed in the management of ischemic diseases. These components consist of five major active compounds: ginsenoside Rg1, ginsenoside Rd, ginsenoside Rb1, ginsenoside Re, and notoginsenoside R1 [12]. Since 1999, the China National Medical Products Administration (NMPA) has approved Xuesaitong soft capsules, formulated from *Panax notoginseng* saponins, for the treatment of IS. This approval is based on its multifaceted pharmacological properties, which include antiplatelet, anticoagulatory, antithrombotic, anti-atherosclerotic, lipid-lowering, vasodilatory, anti-inflammatory, and anti-ischemic effects [13]. A previous pharmacokinetic study has indicated that Xuesaitong can enhance the absorption of aspirin in the gastrointestinal tract, offering additional avenues for combined drug therapies aimed at IS treatment or prevention. Additionally, Xuesaitong has been demonstrated to enhance the expression of vascular endothelial growth factors, which are critical for processes like angiogenesis and endothelial cell proliferation, thereby offering protection against gastrointestinal injury caused by aspirin, as suggested by network pharmacology research [14]. Experimental evidence also shows that Xuesaitong may exert sustained neuroprotective benefits against IS by promoting nerve regeneration, potentially through the inhibition of the ROCKII pathway, induction of an anti-inflammatory M2 microglial phenotype, and downregulation of STAT3 signaling to minimize neuronal apoptosis [15, 16]. Xuesaitong further mitigates IS-associated brain injury by inhibiting intracellular Ca^2+^ overload [17], improving cerebral microperfusion [18], reducing oxidative stress and inflammatory response [19], and stimulating tissue repair through the mobilization of bone marrow mesenchymal stem cells [20].

PANDA study, the largest multicenter, randomized, placebo-controlled clinical trial of Xuesaitong soft capsules, has demonstrated that Xuesaitong could significantly improve the functional independence of AIS patients at three months [21]. Therefore, we conducted this prespecified, secondary analysis (i) to assess improvements in functional outcomes among AIS patients stratified by age into two categories (< 65 and ≥ 65 years) and (ii) determine whether age-specific categories influence the correlation between treatment strategy and functional outcome in the PANDA study.

## Methods

### Study population and design

We performed a multicenter, randomized, double-blind, placebo-controlled trial comprising 3542 patients across 67 medical centers in China between July 1 th, 2018, and June 30 th, 2020. The PANDA trial was registered under the number ChiCTR1800016363 at *28 May 2018, *https://www.chictr.org.cn/showproj.html?proj=26919. The primary aim of the original study was to assess the efficacy and safety of Xuesaitong soft capsules as an adjunct to standard care for patients with AIS. Eligibility criteria for patient inclusion were as follows: 1) age ranging from 18 to 75 years; 2) diagnosed with AIS within 14 days of onset; 3) an mRS score of 0 or 1 prior to the stroke; and 4) an NIHSS score ranging from 4 to 15 at the time of randomization. The comprehensive results of the original study have been previously published [21], and the detailed design and rationale for this prespecified analysis are available in supplementary material. The manuscript was prepared in accordance with the CONSORT reporting guidelines.

An automated centralized system generated the randomization codes, securely stored in sealed, sequential, opaque envelopes. Eligible patients received these codes from their respective participating centers in a strictly sequential manner. Patients were randomly assigned in a 1:1 ratio to either the intervention group, receiving Xuesaitong soft capsules (60 mg per capsule, two capsules administered twice a day), or the placebo group, receiving the Xuesaitong placebo (60 mg per capsule, two capsules administered twice a day) for a period of three months. Both the study drug and the placebo were identically packaged, sharing a uniform appearance and batch number. All participants received standard medical care, comprising antiplatelet therapy and management of vascular risk factors, complied with current guidelines for the diagnosis and management of AIS. In this prespecified analysis, we stratified patients by age, categorizing them into two groups: those under 65 years and those aged 65 years or older. Ethical approval for the study was granted by the ethics committees of all participating centers, and the trial adhered to the ethical principles set forth in the Declaration of Helsinki. The written informed consent was mandatory for all patients before inclusion in the study.

### Definition of study outcomes

The primary outcome for the PANDA study was the proportion of patients who attained functional independence, as indicated by a mRS score of 2 or below at the 3-month follow-up. The mRS operates on an ordinal scale from 0 to 6, where scores from 0 to 1 indicate no disability, scores between 2 and 5 signify varying degrees of disability, and scores of 6 indicates death. To determine this outcome, certified investigators who were blinded to treatment assignments conducted structured interviews during the assessment phase.

The trial also assessed several secondary outcomes: 1) rate of stroke recurrence, encompassing both cerebral infarction and intracerebral hemorrhage (ICH), measured at 3 and 12-month follow-up; 2) percentage of AIS patients achieving functional independence, as characterized by a mRS score of 2 or lower, measured at 12-month follow-up; 3) percentage of participants with no or minimal disability, indicated by mRS scores of 1 or less, measured at both 3 and 12-month follow-up; 4) improvement in neurological deficits, defined by NIHSS score changes from baseline to the 3 month (the NIHSS scale spans from 0 to 42, where higher values reflecting greater stroke severity); 5) incidence of composite cerebrovascular events (CCEs), encompassing cerebral infarction, ICH, myocardial infarction, and vascular mortality, evaluated at the both 3 and 12-month follow-up; 6) quality of life, evaluated using the European Quality of Life Five Dimension (EQ- 5D) index scores at the3 and 12-month follow-up; 7) changes to activities of daily living, evaluated through change in the Barthel Index (BI) from initial assessment to 3 and 12 months. Serious adverse events (SAEs) occurring during a 3-month follow-up period constituted the primary safety outcome. Additional secondary safety outcomes comprised symptomatic ICH, all-cause mortality, and adverse events (AEs) within the initial 3 months.

### Statistical analysis

The dataset adhering to the ITT principle was derived from participants initially enrolled in the study who had received the test drugs at least once and had undergone a minimum of one post-medication evaluation. This ITT population served as the primary cohort for efficacy-data analysis. For safety assessment, the safety analysis set (SAS) comprised the patients administered at least one dose of the study drug.

Age-stratified risk analysis was planned a priori to investigate potential heterogeneity among different age subgroups. The study population was stratified into two predefined age categories: individuals below 65 years and those aged 65 years or older. For continuous variables that follow a normal distribution, patient demographics and clinical features were reported as the mean ± standard deviation (SD). Conversely, for variables not following a normal distribution, medians accompanied by interquartile ranges (IQR) were reported. Categorical variables were presented using numerical values and corresponding percentages. Comparative analyses of the baseline features between the Xuesaitong group and the placebo group within various age subgroups were conducted. The chi-square test was utilized for categorical variables, while Student’s *t*-test or the Mann–Whitney U test was applied for continuous variables.

The Sankey diagram was constructed using the SankeyMATIC online platform, which is freely accessible. To explore the potential nonlinear relationship between age (a continuous variable) and functional independence (the primary outcome), restricted cubic spline curves were employed. To evaluate the therapy effect (Xuesaitong group vs. placebo group) in relation to age stratification (< 65 vs. ≥ 65 years), either the Cox proportional hazard regression model or the logistic regression model was employed, depending on the specific analysis. Results were presented as hazard ratios (HRs) or odds ratios (ORs) with their respective 95% confidence intervals (CIs). Interaction analyses were performed by including a treatment assignment/age interaction term in the model, and the statistical significance of the interaction was assessed. An interaction *p* value below 0.05 was interpreted as evidence that the treatment effect was significantly modified by age. Covariates for adjustment in the analyses were chosen based on previously published literature and clinical relevance, and included factors such as gender, the Trial of Org 101072 in Acute Stroke Treatment (TOAST) classification, initial mRS score, NIHSS score, and any other variables showing baseline differences between groups. These variables were considered in the analyses to mitigate their possible impact on the treatment effect and to placebo for confounding. Furthermore, the interaction between therapy and time post-randomization concerning neurological deficit improvement, quality of life, and changes in activities of daily living was examined employing a generalized linear mixed effects model (GLMM) with repeated measures. Statistical analyses were conducted employing SAS statistical software, version 9.4 (SAS Institute Inc., Cary, NC, USA). Two-sided tests were used with a significance level set at *P* < 0.05.

## Results

### Baseline characteristics

**Fig.1:**
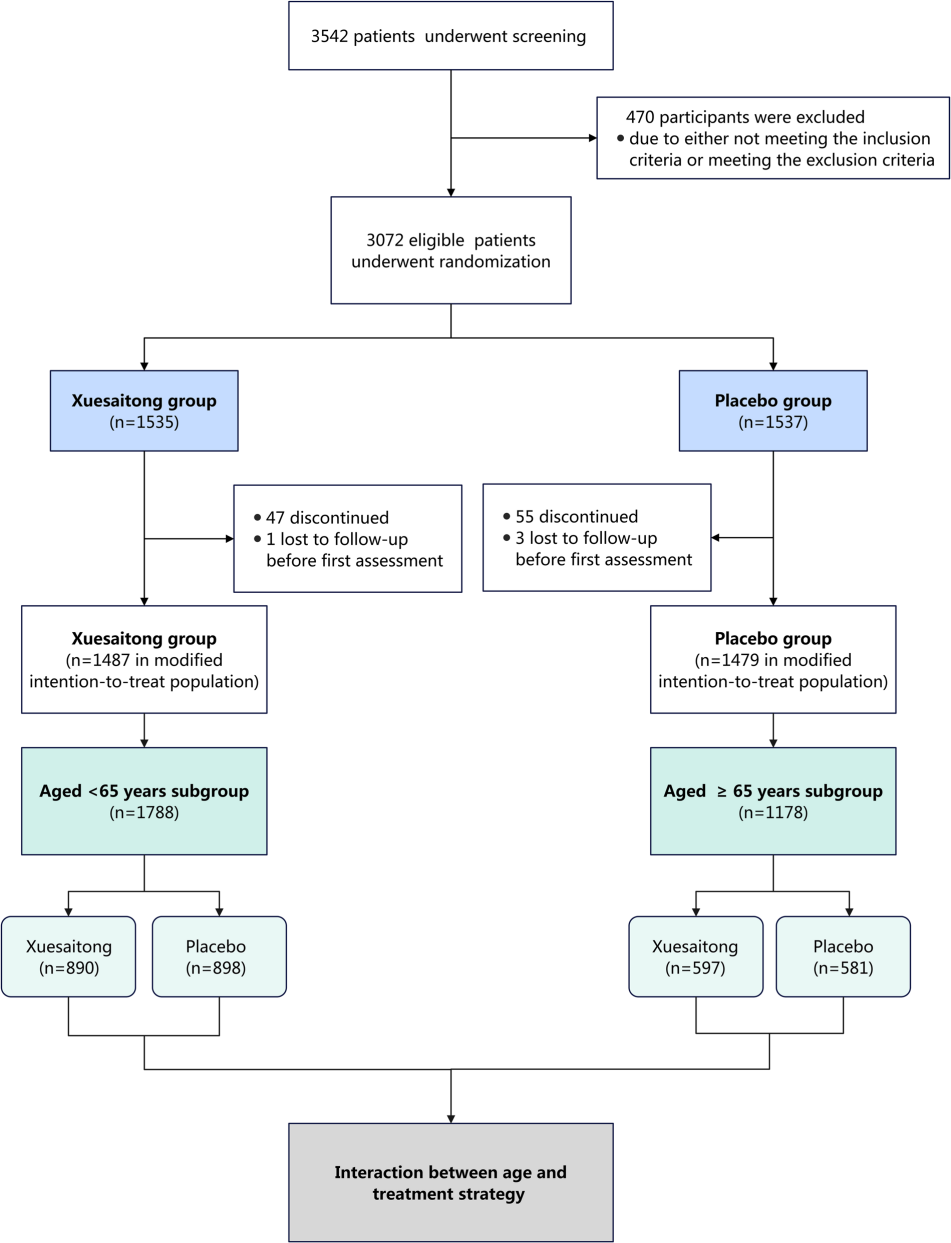
Flowchart of the Study

3072 patients were enrolled from 67 tertiary health centers across China. Following a comprehensive screening process, 2966 patients (1487 in the Xuesaitong group and 1479 in the placebo group) were determined to be eligible and were subsequently included in the ITT dataset. These patients were stratified into two age-based subgroups: 1) 1788 patients (60.28%) were younger than 65 years, and 2) 1178 patients (39.72%) were 65 years or older. The study flowchart is presented in Fig. 1. Patients who were 65 years or older were associated with a reduced proportion of male individuals (57.81% vs. 72.76%) compared to those younger than 65 years. Moreover, patients 65 years or older had a higher likelihood of having diabetes mellitus (25.38% vs. 23.38%), a history of ischemic stroke (17.23% vs. 14.43%), and a classification of IS as large artery atherosclerosis (53.74% vs. 52.80%). Among patients in this older age group, the proportion who had received oral Xuesaitong therapy or antiplatelet agents prior to randomization was greater (1.19% vs. 1.06% and 18.76% vs. 18.51%, respectively) than among those younger than 65 years. The baseline variables were generally well balanced between the Xuesaitong and placebo groups across the age subgroups, with the exceptions of diastolic blood pressure and hypertension in the 65 years or older subgroup, and history of oral Xuesaitong therapy in the younger than 65 years subgroup (Table 1). In the subgroup aged 65 years or older, the median diastolic blood pressure in the placebo group was higher [82 (IQR 77–90) vs. 81 (IQR 75–89), *P* = 0.038] than in the Xuesaitong group. Additionally, the Xuesaitong group had a greater proportion of individuals with a history of hypertension (56.28% vs. 54.91%, *P* = 0.020). Conversely, in the subgroup younger than 65 years, a higher proportion of individuals in the placebo group had received oral Xuesaitong therapy prior to randomization (1.56% vs. 0.56%, *P* = 0.04) compared to the Xuesaitong group.

**Table 1:** Baseline patient characteristics in Xuesaitong and placebo groups by age subgroups

Characteristic	No. (%)					
	< 65 years (n = 1788)			≥ 65 years (n = 1178)		
	Xuesaitong group (n = 890)	Placebo group (n = 898)	P value	Xuesaitong group (n = 597)	Placebo group (n = 581)	P value
Age, median (IQR), y	56 (51–61)	56 (51–61)	0.996	69 (67–72)	69 (67–72)	0.871
Sex						
Male	626 (70.34)	675 (75.17)	0.022	331 (55.44)	350 (60.24)	0.096
Female	264 (29.66)	223 (24.83)		266 (44.56)	231 (39.76)	
BMI^a^, median (IQR), kg/m^2^	24.80 (22.90–27.00)	24.80 (22.90–27.10)	0.404	24.20 (22.35–26.10)	24.00 (21.95–26.00)	0.149
Blood pressure, median (IQR), mmHg						
SBP	143 (132–158)	140 (130–156)	0.150	141 (132–154)	142 (134–158)	0.063
DBP	88 (80–96)	87 (80–96)	0.914	81 (75–89)	82 (77–90)	0.038**
Past medical history						
Hypertension	499 (56.07)	502 (55.90)	0.925	336 (56.28)	319 (54.91)	0.020**
Diabetes mellitus	204 (22.92)	214 (23.83)	0.651	152 (25.46)	147 (25.30)	0.128
Hyperlipidemia	47 (5.28)	51 (5.68)	0.697	26 (4.36)	28 (4.82)	0.285
Ischemic stroke	118 (13.26)	140 (15.59)	0.328	99 (16.58)	104 (17.90)	0.534
Transient ischemic attack	2 (0.22)	7 (0.78)	0.246	1 (0.17)	1 (0.17)	0.477
Smoking status						
Never smoking	431 (48.43)	425 (47.33)	0.967	381 (63.82)	352 (60.59)	0.369
Occasional smoking	66 (7.42)	74 (8.24)		34 (5.70)	33 (5.68)	
Current smoking	271 (30.45)	278 (30.96)		85 (14.24)	107 (18.42)	
Previous smoking	81 (9.10)	81 (9.02)		73 (12.23)	63 (10.84)	
Unknown	41 (4.61)	40 (4.45)		24 (4.02)	26 (4.48)	
History of alcohol use						
Never drinking	474 (53.26)	468 (52.12)	0.555	399 (66.83)	365 (62.82)	0.340
Occasional drinking	192 (21.57)	203 (22.61)		87 (14.57)	107 (18.42)	
Current drinking	137 (15.39)	133 (14.81)		54 (9.05)	48 (8.26)	
Previous drinking	40 (4.49)	54 (6.01)		36 (6.03)	34 (5.85)	
Unknown	47 (5.28)	40 (4.45)		21 (3.52)	27 (4.65)	
TOAST classification^b^						
Large artery atherosclerosis	471 (52.92)	473 (52.67)	0.800	318 (53.27)	315 (54.22)	0.817
Cardioembolism	10 (1.12)	15 (1.67)		5 (0.84)	6 (1.03)	
Small artery occlusion	373 (41.91)	373 (41.54)		245 (41.04)	238 (40.96)	
Stroke of other determined etiology	15 (1.69)	12 (1.34)		8 (1.34)	4 (0.69)	
Stroke of undetermined etiology	21 (2.36)	25 (2.78)		21 (3.52)	18 (3.10)	
History of oral Xuesaitong						
Yes	5 (0.56)	14 (1.56)	0.040**	4 (0.67)	10 (1.72)	0.096
No	885 (99.44)	884 (98.44)		593 (99.33)	571 (98.28)	
History of oral antiplatelet agents						
Yes	165 (18.54)	166 (18.49)	0.977	112 (18.76)	109 (18.76)	1.000
No	725 (81.46)	732 (81.51)		485 (81.24)	472 (81.24)	
mRS^c^						
mRS ≤ 1	282 (31.69)	330 (36.75)	0.075	218 (36.52)	208 (35.80)	0.716
1 < mRS ≤ 2	193 (21.69)	185 (20.60)		114 (19.10)	122 (21.00)	
3 ≤ mRS ≤ 5	415 (46.63)	383 (42.65)		265 (44.39)	251 (43.20)	
NIHSS^d^ score, median (IQR)	5 (4–7)	5 (4–7)	0.264	5 (4–7)	5 (4–7)	0.134
EQ- 5D^e^ score, median (IQR)	75 (60–88)	75 (60–89)	0.764	70 (60–85)	75 (60–90)	0.458
BI^f^, median (IQR)	75 (55–90)	80 (55–90)	0.053	75 (55–90)	75 (55–90)	0.602

NIHSS, National Institutes of Health Stroke Scale; EQ- 5D, EuroQoL- 5 dimensions; BMI, Body Mass Index; BI, Barthel Index; mRS, modified Rankin Scale; SD, standard deviation; SBP, systolic blood pressure; DBP, diastolic blood pressure. Data are presented as mean (SD), n (%), or median with interquartile ranges (IQR) in all included participants

^a^BMI was calculated as weight in kilograms divided by height in meters squared

^b^The Trial of Org 10,172 in Acute Stroke Treatment (TOAST) classification denotes five subtypes of ischemic stroke: 1) large artery atherosclerosis, 2) cardioembolism, 3) small artery occlusion, 4) stroke of other determined etiology, and 5) stroke of undetermined etiology

^c^mRS is an ordinal scale ranging from 0 to 6, with scores 0–1 representing no disability, scores 2–5 indicating increasing levels of disability, and scores 6 indicating death

^d^NIHSS score indicated the severity of neurological deficits, ranging from 0 to 42, with higher scores indicating more severe strokes

^e^EQ- 5D score referred to 5 dimensions that describe health states, including mobility, usual activities, self-care, feelings of pain or discomfort, feelings of anxiety or depression

^f^BI was an ordinal scale which measured a person's ability to complete activities of daily living (ADL)

**The difference between groups as statistically significant (P value < 0.05)

### Interaction between age and treatment strategy for the efficacy outcomes

**Fig.2:**
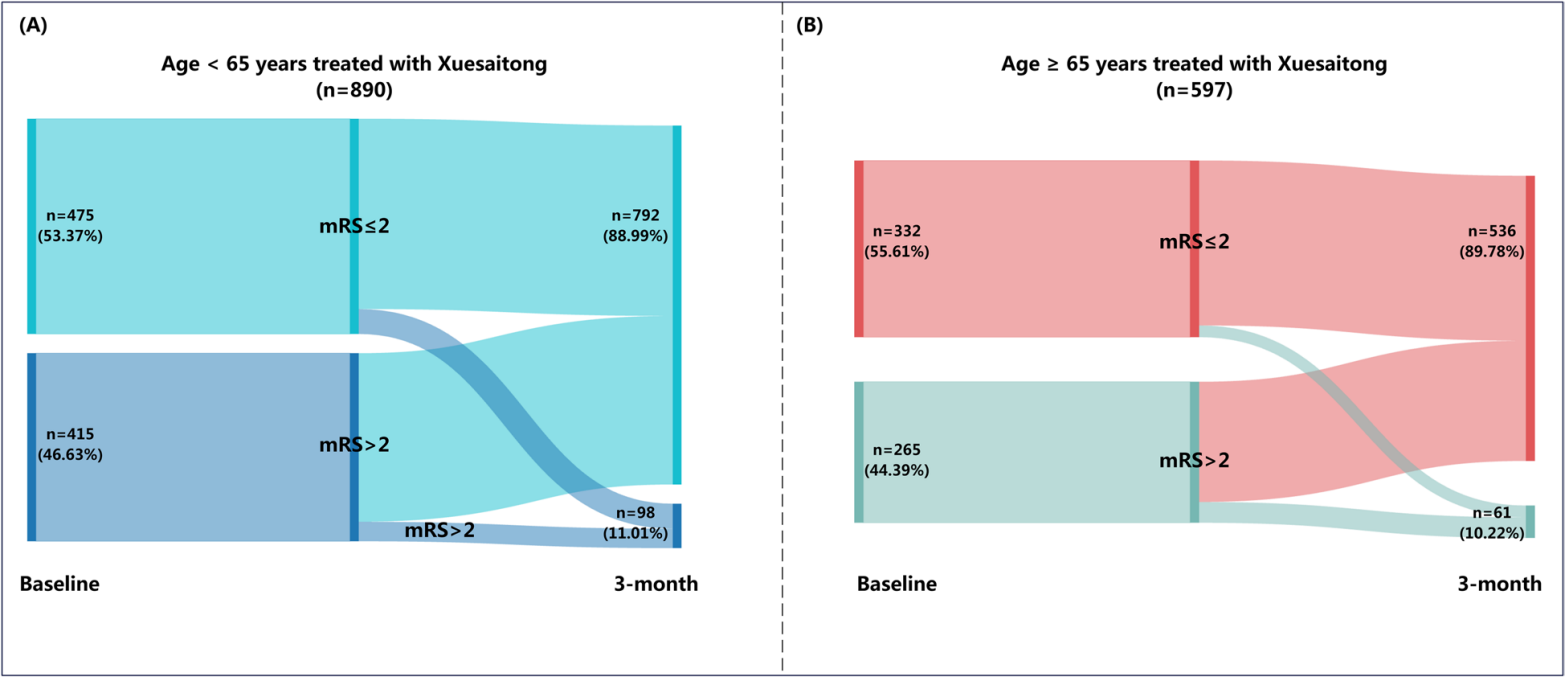
Change in the proportion of functional independence from baseline to 3-month follow-up. mRS, modified Rankin Scale. **A** Sankey diagrams depicting the change in functional independence (defined as a mRS of 0 to 2) of patients treated with Xuesaitong in age < 65 strata. **B** Sankey diagrams depicting the change in functional independence of patients treated with Xuesaitong in age ≥ 65 strata

**Fig. 3:**
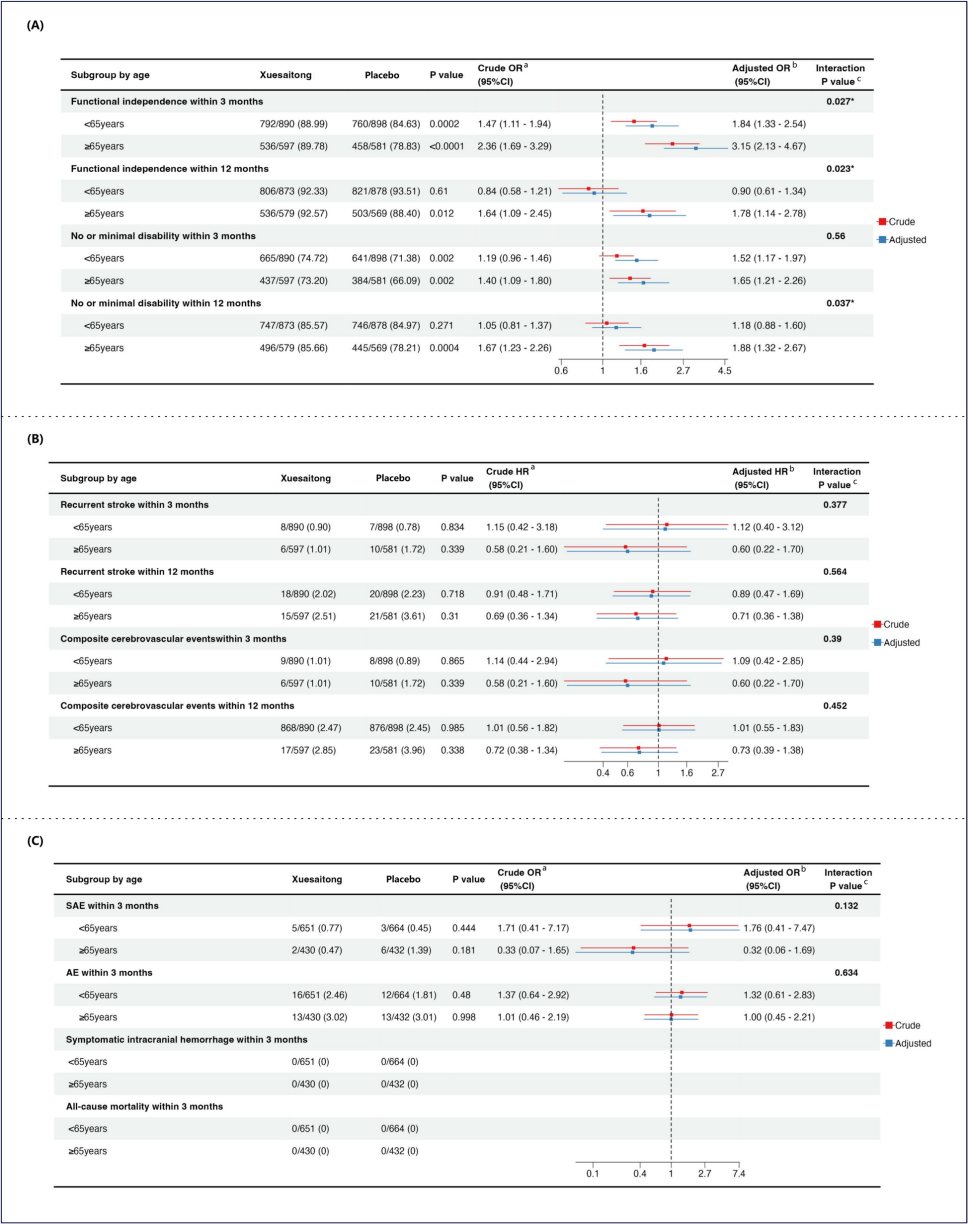
Forest plot for primary efficacy outcome, secondary efficacy outcomes and safety outcomes, stratified by age. OR, odds ratio; HR, hazard ratio; SAE, severe adverse event; AE, adverse event; The effect of Xuesaitong therapy compared with placebo therapy on primary, secondary efficacy and safety outcomes were described in terms of event rates, HRs, ORs, and interaction terms. ^a^ Logistic or cox regression analysis was applied to estimate crude OR/HR. ^b^ Adjusted OR/HR was consequently estimated by adjusting the crude OR/HR for gender, the TOAST classification, baseline mRS score, baseline NIHSS score, and any other variables that displayed baseline inter-group differences. ^c^ Interaction P value was for the interaction between treatment and subgroup. A significant p-interaction value (< 0.05) indicated that the treatment effect was influenced by age. **A** Forest plot for functional independence (defined as a mRS of 0 to 2) and no or minimal disability (defined as a mRS of 0 to 1) within 3 and 12 months. **B** Forest plot for recurrent stroke and composite cerebrovascular events (including cerebral infarction, intracerebral hemorrhage, myocardial infarction, and vascular death), measured at 3 and 12 months. **C** Forest plot for safety outcomes within 3 months

As illustrated by the Sankey diagrams, significant improvements in functional independence from baseline to 3 months were observed in both age categories when treated with Xuesaitong (Fig. 2). Specifically, among participants aged 65 years or older, 536 (89.78%) out of 597 patients randomly assigned to Xuesaitong achieved functional independence within 3 months, compared to 458 (78.83%) out of 581 patients assigned to placebo (adjusted odds ratio (aOR): 3.15; 95% CI: 2.13–4.67; *P* < 0.0001). In the group younger than 65 years, 792 (88.99%) out of 890 patients assigned to Xuesaitong achieved functional independence within 3 months, as opposed to 760 (84.63%) out of 898 patients assigned to placebo (aOR: 1.84; 95% CI: 1.33–2.54; *P* = 0.0002). Age-stratified analyses revealed heterogeneity in the rate of achieving functional independence within 3 months between Xuesaitong and placebo treatments (*P* = 0.027 for interaction) (Fig. 3A).

Among patients aged 65 and older, the Xuesaitong group exhibited a significantly greater proportion of functional independence at the 12-month follow-up in comparison to the placebo group (92.57% vs. 88.40%; aOR: 1.78, 95% CI: 1.14–2.78, *P* = 0.012). Conversely, for patients under 65 years, no significant difference was detected in the proportion of functional independence at the 12-month follow-up between the Xuesaitong and placebo groups (92.33% vs. 93.51%; aOR: 0.90, 95% CI: 0.61–1.34, *P* = 0.61). A significant interaction between categorical age and treatment was observed, indicating that the influence of Xuesaitong on functional independence at 12 months varied by age (*P* = 0.023 for interaction). The percentage of AIS patients with no or minimal disability at the 3-month follow-up was similar between the Xuesaitong and placebo groups in both age categories (< 65 years, aOR: 1.52, 95% CI: 1.17–1.97, *P* = 0.002; ≥ 65 years, aOR: 1.65, 95% CI: 1.21–2.26, *P* = 0.002; *P* = 0.56 for interaction). In patients aged 65 and older, the Xuesaitong group demonstrated a significantly higher proportion of no or minimal disability at 12 months compared to the placebo group (85.66% vs. 78.21%; aOR: 1.88, 95% CI: 1.32–2.67, *P* = 0.0004). However, among patients under 65 years, the proportion of no or minimal disability at 12 months was not significantly different (85.57% vs. 84.97%; aOR: 1.18, 95% CI: 0.88–1.60, *P* = 0.271). Age-stratified analyses demonstrated heterogeneity in the proportion of no or minimal disability at 12 months when comparing Xuesaitong to placebo (*P* = 0.037 for interaction) (Fig. 3A).

In the analysis of recurrent stroke incidence within 3 and 12 months, no notable difference was detected between the Xuesaitong group and the placebo group across both age strata [(< 65 years within 3 months, aOR: 1.12, 95% CI: 0.40–3.12, *P* = 0.834; ≥ 65 years within 3 months, aOR: 0.60, 95% CI: 0.22–1.70, *P* = 0.339; *P* for interaction = 0.377); (< 65 years within 12 months, aOR: 0.89, 95% CI: 0.47–1.69, *P* = 0.718; ≥ 65 years within 12 months, aOR: 0.71, 95% CI: 0.36–1.38, *P* = 0.31; *P* for interaction = 0.564)]. Similarly, no significant disparity was found in the incidence of CCEs at 3-month and 12-month intervals for both age categories [(< 65 years within 3 months, aOR: 1.09, 95% CI: 0.42–2.85, *P* = 0.865; ≥ 65 years within 3 months, aOR: 0.60, 95% CI: 0.22–1.70, *P* = 0.339; *P* for interaction = 0.39); (< 65 years within 12 months, aOR: 1.01, 95% CI: 0.55–1.83, *P* = 0.985; ≥ 65 years within 12 months, aOR: 0.73, 95% CI: 0.39–1.38, *P* = 0.338; *P* for interaction = 0.452)] (Fig. 3B).

Table 2 demonstrates advancements in neurological deficits, quality of life, and activities of daily living during the follow-up period. In terms of the NIHSS score change from initial assessment to 3 months, no significant difference was noted between the Xuesaitong and placebo groups in either age category [< 65 years: − 4 (IQR, − 5 to − 3) vs. − 4 (IQR, − 5 to − 3), *P* = 0.130; ≥ 65 years: − 4 (IQR, − 5 to − 3) vs. − 4 (IQR, − 5 to − 3), *P* = 0.083]. For patients aged ≥ 65 years, the EQ- 5D scores at the 3-month [90 (IQR, 80 to 95) vs. 90 (IQR, 80 to 95), *P* = 0.008) and 12-month follow-up [95 (IQR, 90 to 98) vs. 90 (IQR, 90 to 95), *P* = 0.010] were markedly elevated in the Xuesaitong group compared to the placebo group. There was no significant interaction between follow-up time and therapy (*P*_int_ = 0.272). Among patients aged < 65 years, no notable differences in EQ- 5D scores were found at 3 months [90 (IQR, 80 to 95) vs. 90 (IQR, 80 to 95), *P* = 0.524] or 12 months [95 (IQR, 90 to 98) vs. 95 (IQR, 90 to 96), *P* = 0.200] between the Xuesaitong and placebo groups. No heterogeneity was noted in quality-of-life analyses over time (*P*_int_ = 0.870). Likewise, BI changes from initial assessment to 3 and 12 months did not differ significantly for patients under 65 years [3 months: 15 (IQR, 5 to 35) vs. 15 (IQR, 5 to 30), *P* = 0.052; 12 months: 20 (IQR, 5 to 40) vs. 20 (IQR, 5 to 40), *P* = 0.214], with no notable interaction between follow-up time and treatment (*P*_int_ = 0.553). In contrast, for patients aged ≥ 65 years, a significant BI increase from initial assessment to 3 months was observed in the Xuesaitong group compared to the placebo group [20 (IQR, 5 to 35) vs. 15 (IQR, 5 to 30), *P* = 0.045]. However, no difference was noted at 12 months [25 (IQR, 5 to 40) vs. 20 (IQR, 5 to 40), *P* = 0.527], and no interactions between follow-up time and therapy were significant.

**Table 2:** Secondary Efficacy Outcomes in Xuesaitong and Placebo Groups by Age Subgroup

Outcomes	Age < 65 years				Age ≥ 65 years			
	Xuesaitong group	Placebo group	P value*	Interaction effect^a^ (Time*Group)	Xuesaitong group	Placebo group	P value*	Interaction effect^a^ (Time*Group)
NIHSS^b^ score								
Score within 3 mo, median (IQR)	1 (0 to 3)	1 (0 to 3)	0.562	NA	1 (0 to 3)	2 (0 to 3)	0.004**	NA
Score change from baseline to 3 mo, median (IQR)	− 4 (− 5 to − 3)	− 4 (− 5 to − 3)	0.130	NA	− 4 (− 5 to − 3)	− 4 (− 5 to − 2)	0.083	NA
EQ- 5D^c^ score								
Score within 3 mo, median (IQR)	90 (80 to 95)	90 (80 to 95)	0.524	0.870	90 (80 to 95)	90 (80 to 95)	0.008**	0.272
Score within 12 mo, median (IQR)	95 (90 to 98)	95 (90 to 96)	0.200		95 (90 to 98)	90 (90 to 95)	0.010**	
BI^d^ change								
Score change from baseline to 3 mo, median (IQR)	15 (5 to 35)	15 (5 to 30)	0.052	0.553	20 (5 to 35)	15 (5 to 30)	0.045**	0.620
Score change from baseline to 12 mo, median (IQR)	20 (5 to 40)	20 (5 to 40)	0.214		25 (5 to 40)	20 (5 to 40)	0.527	

BI, Barthel Index; EQ- 5D, EuroQoL Group 5-Dimension; NA, not applicable; NIHSS, National Institutes of Health Stroke Scale; Data are presented as median with interquartile ranges (IQR) of patients unless otherwise indicated

*Wilcoxon rank-sum test was applied. The tests were 2-sided. P value of less than 0.05 was considered significant

^a^An interaction P value less than 0.05 indicated that the treatment effect was significantly influenced or modified by time

^b^NIHSS score indicated the severity of neurological deficits, ranging from 0 to 42, with higher scores indicating more severe strokes

^c^EQ- 5D score referred to 5 dimensions that describe health states, including mobility, usual activities, self-care, feelings of pain or discomfort, feelings of anxiety or depression

^d^BI was an ordinal scale which measured a person’s ability to complete activities of daily living (ADL)

**The difference between groups as statistically significant (P value < 0.05)

### Interaction between age and treatment strategy for safety outcomes

A total of 2177 patients comprised the SAS, with 1315 (60.40%) and 862 (39.60%) patients belonging to age subgroups of < 65 years and ≥ 65 years, respectively. For the primary safety outcome, namely SAEs within 3 months, no significant differences were detected between the Xuesaitong and placebo groups in either age subgroup (< 65 years, aOR: 1.76, 95% CI: 0.41–7.47, *P* = 0.444; ≥ 65 years, aOR: 0.32, 95% CI: 0.06–1.69, *P* = 0.181; *P* = 0.132 for interaction). Secondary safety outcomes, including symptomatic ICH, all-cause mortality, and AEs within 3 months, also indicated no significant differences between the two groups, irrespective of age. No age-treatment interaction was noted when comparing patients who treated with Xuesaitong to those given placebo therapy (Fig. 3C).

### Age-dependent effect of treatment strategy

**Fig. 4:**
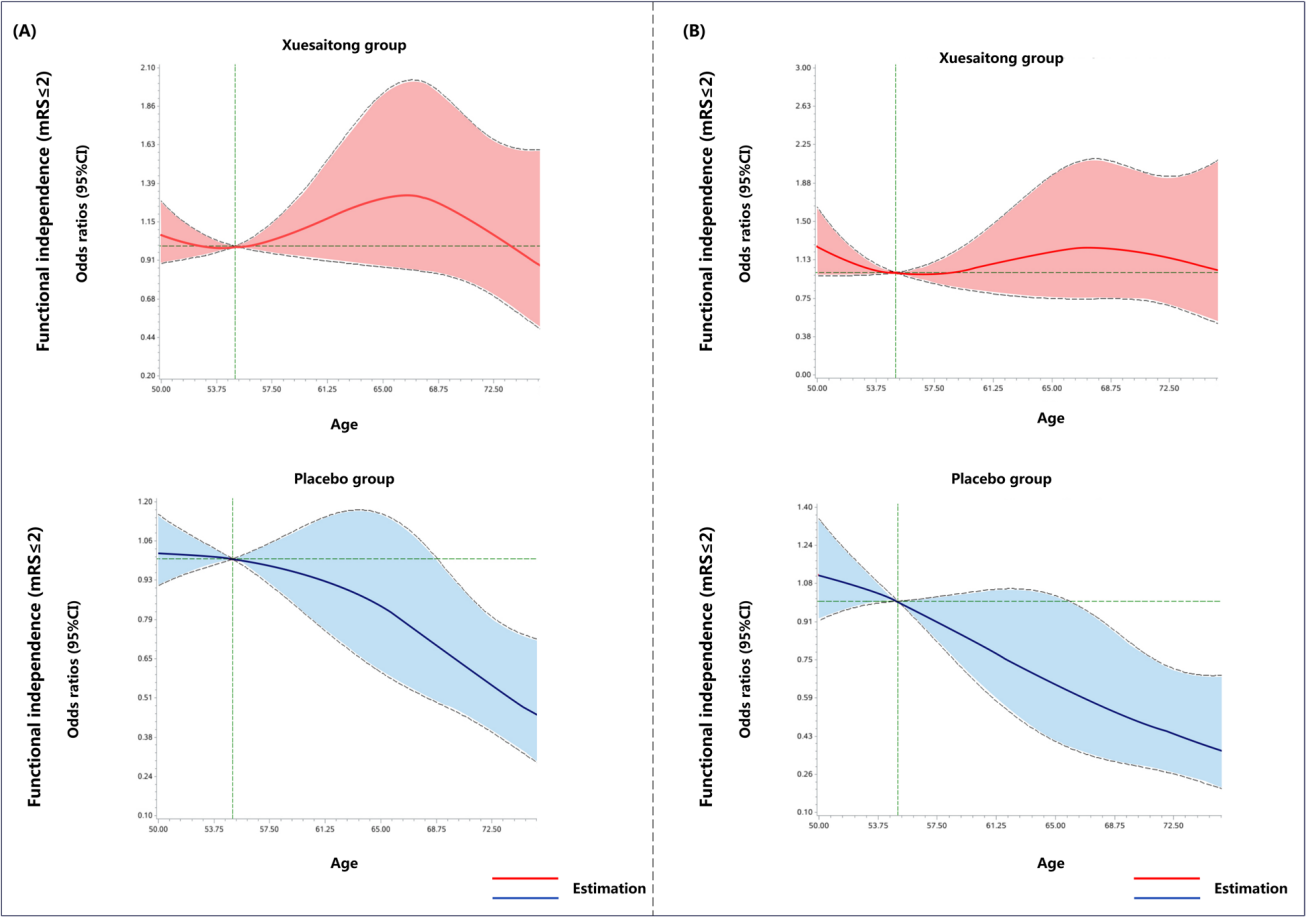
Association between age and short and long-term functional independence, delineated by restricted cubic splines. Abbreviations: mRS, modified Rankin Scale. **A** Association between age and short-term functional independence (defined as a mRS of 0 to 2) within 3 months in Xuesaitong group and placebo group. **B** Association between age and long-term functional independence within 12 months in Xuesaitong group and placebo group

Figure 4 uses restricted cubic splines to illustrate dynamic age-dependent trends in functional independence across two treatment strategies at 3 and 12 months. In the placebo group effect pattern (Fig. 4A), the event rate curve for functional independence within 3 months decreased as age increased, indicating that the geriatric population with AIS found it challenging to derive substantial benefits from standard care. A similar trend was evident for functional independence within 12 months (Fig. 4B). In contrast, the Xuesaitong group’s event rate curve initially increased and then decreased with age, suggesting an age-dependent benefit of Xuesaitong therapy on short- and long-term functional independence, except in the very older population. It appeared that as patients reached an advanced age accompanied by a decline in physical functionality, the effectiveness of Xuesaitong therapy was less pronounced. Overall, the event rate curve for the treatment group was higher than that for the placebo group, indicating that Xuesaitong therapy was superior to placebo in improving functional independence, irrespective of age.

## Discussion

Advanced age serves as an independent predictor of both bleeding and ischemic events [22–24] and remains the most unmodifiable risk factor for stroke events. Despite this, clinical data on therapies for IS in the older population (aged ≥ 65 years) are limited. Our age-stratified prespecified analysis revealed that functional independence in older patients (aged ≥ 65 years) aligned with the overall results of our previously published trial [21]. Moreover, the effects of Xuesaitong therapy on functional independence, both in the short and long term, was more pronounced in older patients aged ≥ 65 years, exhibiting a notable interaction between categorical age and treatment strategy.

### Interpretation of the results

In our analysis focusing on older patients aged ≥ 65 years, Xuesaitong therapy was associated with a notably higher probability of attaining functional independence [aOR_3-month_: 3.15, 95% CI: 2.13–4.67,* P* < 0.0001; aOR_12-month_: 1.78, 95% CI: 1.14–2.78,* P* = 0.012] and no or minimal disability [aOR_3-month_: 1.65, 95% CI: 1.21–2.26, *P* = 0.002; aOR_12-month_: 1.88, 95% CI: 1.32–2.67,* P* = 0.0004] over both short- and long-term periods, in comparison to the placebo group. Conversely, in patients aged < 65 years, Xuesaitong therapy showed a significantly higher rate of functional independence [aOR_3-month_: 1.84, 95% CI: 1.33–2.54,* P* = 0.0002] and no or minimal disability [aOR_3-month_: 1.52, 95% CI: 1.17–1.97,* P* = 0.002] at 3 months but did not maintain these benefits in long-term follow-up [aOR _functional independence at 12-month_: 0.90, 95% CI: 0.61–1.34,* P* = 0.61; aOR _no or minimal disability at 12-month_: 1.18, 95% CI: 0.88–1.60,* P* = 0.271]. In terms of stroke recurrence and CCEs at 3 or 12 months, Xuesaitong therapy did not show a significant difference compared to the placebo group, irrespective of age. Importantly, our subgroup analysis included an interaction test, a crucial step for identifying subgroup effects. An interaction test *p*-value below 0.05 signifies a significant effect within subgroups; the less the *p* value, the more pronounced the effect. Subgroup effects fall into two categories: those relating to efficacy magnitude and those concerning the nature of the effect. Our interaction test results suggested that Xuesaitong therapy for AIS may offer greater clinical benefits specifically for the older population.

Despite the original study indicating that Xuesaitong was linked to significant improvements in neurologic deficits, no substantial difference was found between Xuesaitong and placebo in neurological deficit improvement (measured by NIHSS score change from baseline) across both age categories. Interestingly, a significant decrease in NIHSS score within 3 months in the older patients aged ≥ 65 years was detected in the Xuesaitong group in comparison to the placebo group; however, this finding not replicated in non-elderly patients. This could be attributed to the enrollment of the non-elderly group under less severe conditions. Regarding quality of life and improvements in activities of daily living, Xuesaitong was linked to a significant and consistent increase in EQ- 5D scores and BI changes in the older population but not in non-elderly patients. Collectively, the present prespecified study suggests that Xuesaitong may yield more favorable outcomes in improving neurologic deficits, quality of life, and activities of daily living specifically among older individuals aged ≥ 65 years.

Safety is a critical factor in the clinical use of pharmaceuticals, particularly for geriatric patients. Stroke prevention in the older patients is complex for several reasons. First, older individuals are often underrepresented in RCTs, resulting in limited age-specific data [25]. Second, the etiology of stroke changes with age, adding complexity to preventive treatment strategies. Lastly, the existing literature in geriatric medicine can offer conflicting views on conventional stroke prevention methods, including issues such as frailty, polypharmacy, and fall risks [26]. To understand the potential age-related treatment variations, it is crucial to investigate any evidence of age-related interactions with the treatment being studied. In our analysis, Xuesaitong therapy showed comparable rates of 3-month SAEs and AEs to the placebo group, regardless of age. Neither symptomatic intracranial hemorrhage nor all-cause mortality was observed in either age group for both treatment modalities.

### Significance of the study and comparison with relevant research

The crucial role of antiplatelet therapy in preventing recurrent stroke among patients with transient ischemic attack or IS is well-established. However, of the available antiplatelet agents, only aspirin has been studied during the acute stage of IS, with its effectiveness considered modest [27, 28]. Prior post hoc analyses have suggested that the benefits of clopidogrel are amplified in high-risk subgroups, including the individuals with a history of IS, myocardial infarction [29], diabetes mellitus [30], cardiac surgery [31], or those receiving lipid-lowering treatments. Nonetheless, considerable interindividual variability exists in the antiplatelet response to clopidogrel. Substantial evidence links advanced age with a higher incidence of high platelet reactivity (HPR) in patients given clopidogrel [32–34], and those exhibiting HPR carry an elevated risk for recurrent thrombotic events, often referred to as “clopidogrel resistance” [35, 36]. Therefore, additional research is needed to explore personalized treatment strategies for AIS patients, especially those of advanced age.

A previous study that included 1,168 patients evaluated the effectiveness of edaravone dexborneol in treating AIS. This study found comparable enhancements in neurological performance for both older and non-elderly AIS patients. However, a prespecified analysis of the primary outcome (mRS ≤ 1) at 3 months showed no age-related efficacy compared to a control group [37]. Notably, in that study, edaravone was employed as the positive control medication, and patients with NIHSS scores spanning from 4 to 24, who could receive the investigational drugs within 48 h after the onset of symptoms, were included. The discrepancies between these findings and ours could be attributed to differences in study design, timing of drug intervention, or choice of control drugs. Moreover, the effectiveness of endovascular thrombectomy (ET) in octogenarians with AIS is still a subject of debate. A multicenter study in real-world conditions found significantly lower proportion of functional independence in older patients following ET in comparison to younger individuals [38]. Therefore, our findings suggest that Xuesaitong may hold potential as a safe and effective alternative therapy for improving the prognosis of older AIS patients.

### Mechanistic insights into Xuesaitong therapy for the older IS population

Our findings suggest that Xuesaitong soft capsules play a pivotal role in mitigating the inflammatory reactions associated with AIS in elderly patients. Existing evidence points to inflammation as a contributing risk factor for cardiocerebrovascular diseases [39]. A growing body of studies has explored the relationship between aging and the inflammatory responses implicated in AIS [40, 41]. Data from the HIBISCUS-STROKE cohort study indicated that patients aged 65 years and older have a unique systemic inflammatory profile compared to those younger than 65 years. Specifically, elevated levels of IL- 6 and sTNF-RI were observed in older AIS patients within the initial 48 h following a stroke [42]. IL- 6, in particular, is instrumental in thrombo-inflammatory processes and correlates with reperfusion failure in AIS [43, 44].

Components derived from traditional medicine have shown promising characteristics, including antioxidative and anti-inflammatory properties, suggesting their potential utility in treating age-related diseases [45]. For example, ginsenoside Rb1, a principal therapeutic component of Xuesaitong, has been demonstrated to preserve the integrity of the blood–brain barrier in ischemic stroke. This preservation occurs through the inhibition of matrix metalloproteinase- 9 (MMP- 9) and nicotinamide adenine dinucleotide phosphate oxidase 4 (NOX4)-generated free radicals induced by neuroinflammation [46]. Additionally, ginsenoside Rb1 has been found to attenuate the activation of ischemic penumbra microglia, as evidenced by the downregulation of TNF-α and IL- 6 expression [47]. This suggests that ginsenoside Rb1 could assist in rescuing the ischemic penumbra by inhibiting microglia-induced neuroinflammation. Furthermore, *Panax notoginseng* has demonstrated potential for exerting anti-brain-aging effects in diseases induced by brain aging, including stroke, Alzheimer’s disease, and Parkinson’s disease. These effects are likely due to their various pharmacological activities, encompassing antioxidation, lipid-lowering, and prevention of vascular remodeling [48]. However, additional research is essential to clarify the precise mechanism by which Xuesaitong could benefit elderly AIS patients.

### Strengths and limitations of the study

This study has several strengths worth noting. Importantly, the subgroup analysis carried out was prespecified rather than being a post hoc analysis. Post hoc analyses, in which hypotheses are not defined prior to evaluation, may introduce substantial concerns owing to uncertainty about the number of analyses performed and the risk of data-driven motivations influencing results [49]. To enhance the credibility and reliability of our findings, we prespecified our subgroup hypotheses before data collection. Moreover, we observed consistent improvements in functional independence due to Xuesaitong compared to the placebo group, throughout the whole population and among predefined age categories. Additionally, we explored indirect evidence to offer potential mechanistic insights, which lends further biological plausibility to the interaction effects observed.

However, some limitations should be considered. First, the study sample consisted solely of Chinese patients, with a male predominance and fewer cases of cardioembolism, which calls into question the external generalizability of our sub-analysis findings and necessitates further validation. Second, despite conducting an interaction test to assess differences in treatment effects between age subgroups, thereby addressing issues of multiplicity and inflated significance levels, the *p* value for the interaction test was not sufficiently small to confirm strong subgroup effects [50]. Moreover, we recognize that the inclusion criteria of patients aged between 18 and 75 years could be a significant limitation. Although our study’s substantial sample size somewhat mitigates sample size disparities between the two age subgroups, it remains crucial to corroborate our findings through a rigorously designed study involving a larger cohort of senior patients. Finally, it is essential to note that most IS patients in this study did not receive intravenous thrombolysis or mechanical thrombectomy. Therefore, further research is required to assess the efficacy and safety of agent post-successful recanalization in older patients. This will facilitate a better understanding the potential benefits and risks, ultimately enhancing stroke management in this vulnerable population.

## Conclusion

In conclusion, this age-stratified, prespecified analysis suggests that Xuesaitong may offer potential benefits in improving functional independence in AIS patients, irrespective of age. Notably, in the PANDA trial, individuals aged 65 and older appear to derive more substantial clinical benefits from Xuesaitong therapy for AIS. Importantly, these improvements occur without a concomitant increase in the risk of SAEs. The conclusion needs further validation to enable a more precise assessment of efficacy, ultimately aiding in the formulation of more effective treatment modalities that are appropriately tailored to diverse age categories, thereby optimizing clinical outcomes for a broader patient population.

## Data Availability

The datasets generated and/or analyzed during the current study are available from the corresponding author on reasonable request.

## Abbreviations

AIS: Acute ischemic stroke
AEs: Adverse events
BI: Barthel Index
CCEs: Composite cerebrovascular events
CIs: Confidence intervals
EQ- 5D: European Quality of Life Five Dimension
GLMM: Generalized linear mixed effects model
HRs: Hazard ratios
ICH: Intracerebral hemorrhage
IQR: Interquartile ranges
IS: Ischemic stroke
ITT: Intention-to-treat
MMP- 9: Matrix metalloproteinase- 9
MRS: Modified Rankin Scale score
NMPA: National Medical Products Administration
ORs: Odds ratios
SAEs: Serious adverse events
SAS: Safety analysis set
SD: Standard deviation
TOAST: Trial of Org 101072 in Acute Stroke Treatment
